# Persistent ferroptosis promotes cervical squamous intraepithelial lesion development and oncogenesis by regulating KRAS expression in patients with high risk-HPV infection

**DOI:** 10.1038/s41420-022-01013-5

**Published:** 2022-04-14

**Authors:** Tianming Wang, Min Gong, Yuting Cao, Chengcheng Zhao, Yingfei Lu, Yu Zhou, Shasha Yao, Jianquan Chen, Chun Zhao, Rong Ju

**Affiliations:** 1 Central Laboratory, Translational Medicine Research Center, The Affiliated Jiangning Hospital with Nanjing Medical University, Nanjing, Jiangsu China; 2 Department of Obstetrics and Gynecology, The Affiliated Jiangning Hospital with Nanjing Medical University, Nanjing, Jiangsu China; 3 Nanjing Medical University, Nanjing, Jiangsu China; 4 Department of Clinical Laboratory, The Affiliated Jiangning Hospital with Nanjing Medical University, Nanjing, Jiangsu China; 5 State Key Laboratory of Reproductive Medicine, Department of Reproduction, Women’s Hospital of Nanjing Medical University, Nanjing Maternity and Child Health Care Hospital, Nanjing, Jiangsu China

**Keywords:** Cervical cancer, Cell proliferation, Apoptosis

## Abstract

Cervical squamous cell carcinoma (CSCC) is a type of female cancer that affects millions of families worldwide. Human papillomavirus (HPV) infection is the main reason for CSCC formation, and squamous intraepithelial lesions (SILs) induced by high-risk HPV (HR-HPV) infection are considered precancerous lesions. A previous study reported that HPV-infected cancer cells were able to counteract lipid peroxidation for survival. Recent research has reported that ferroptosis acts in an iron-dependent lipid peroxidation manner to kill cancer cells, and it is proposed as a new approach for female cancer therapy. Here, we investigated the role of ferroptosis throughout SIL development into CSCC. We found that ferroptosis occurred in SIL, but anti-ferroptosis emerged in CSCC. Our data further indicated that an antiferroptotic effect was formed in response to persistent ferroptosis and then promoted oncogenesis. Altogether, we provide novel insight into ferroptosis in cervical SIL development and suggest a potential therapeutic target for the treatment of CSCC.

## Introduction

Cervical cancer is one of the most common female cancers, with more than 50,000 new cases per year worldwide [1]. The initial formation of cervical squamous cell carcinoma (CSCC), a type of cervical cancer, is associated with single or multiple specific types of human papillomavirus (HPV) infection [2–4]. High-risk HPV (HR-HPV) infection leads to early dysplasia, such as endocervicitis and squamous intraepithelial lesion (SIL). If dysplasia is unable to recover, it develops from a low-grade squamous intraepithelial lesion (LSIL) into a high-grade squamous intraepithelial lesion (HSIL), and most HSIL will further develop into squamous cell carcinoma (SCC) [5]. Understanding the physiopathological mechanism of SIL development and SCC initial emergence may lead to the development of new approaches for the diagnosis and clinical therapy of cervical cancer.

In cancer research, ferroptosis and other regulated cell death (RCD) types, including apoptosis, pyroptosis and necroptosis, are the most relevant RCD modalities reported in the context of cancer [6, 7]. Ferroptosis is characterized by iron accumulation and lipid peroxidation that ultimately induce oxidative damage [8, 9]. In the observation of the subcellular fraction, the number of mitochondrial inner cristae was reduced or even absent, and the density of the outer mitochondrial membrane was decreased and ruptured [6]. Among the factors regulating ferroptosis, GPX4 exerts an anti-ferroptotic effect that defends against excessive lipid peroxidation by reducing phospholipid hydroperoxide to hydroxyphospholipid [10]. Previous studies have reported that inducing ferroptosis by inhibiting intracellular GPX4 is a potential approach for the treatment of cancers in women [11]. Ferroptosis was recently reported to have dual functions: ferroptosis acts not only as a defense mechanism, but can also promote oncogenesis [12, 13]. HPV-related cancer cells have the ability to counteract lipid peroxidation [14]. Although ferroptosis is proposed as a useful therapeutic strategy in several other cancers, its role in the progression of the SIL to SCC transition with HR-HPV infection is poorly understood [15–17].

Here, we verified whether ferroptosis occurred throughout the pathologic progression from SIL to SCC and investigated the underlying mechanism using cervical cancer cell lines. We demonstrated that ferroptosis occurred in LSILs, and persistent ferroptosis induced some lesion cells to present anti-ferroptotic effects and thereby promoted oncogenesis. Overall, our study provides important insights into the effect of ferroptosis on cervical SIL development progression and the results strongly suggest that the characteristics of ferroptosis can be utilized for CSCC treatment.

## Results

### High-risk HPV persistent infection facilitates squamous intraepithelial lesion development

**Fig. 1:**
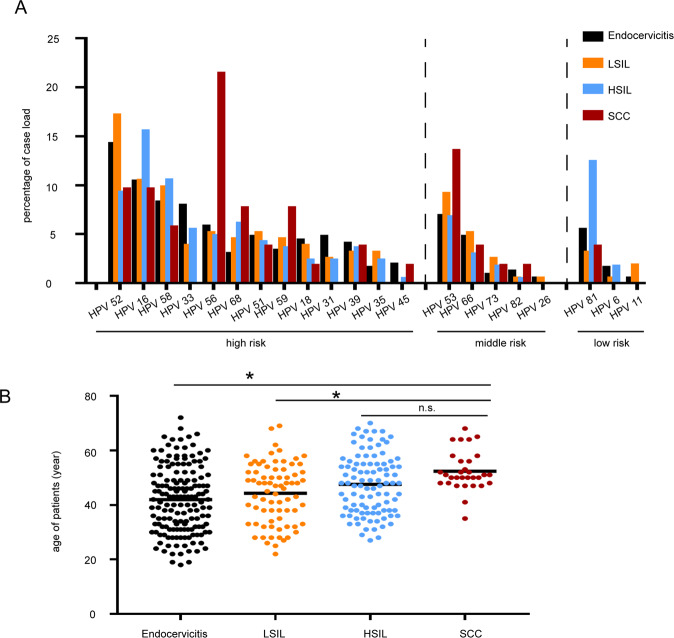
Persist HR-HPV infection is associated with SIL development. Patients data were collected from 2016 to 2020 and the cervical specimens were separated by histopathology into four disease stages: endocervicitis, LSIL, HSIL, and SCC. **A** Infection of different HPV types in patients through SIL development. **B** Distribution of ages in patients through SIL development.

To explore the underlying mechanism that affects SIL development progression, we first reviewed the distribution of different types of HPV infection in squamous intraepithelial lesion (SIL) and squamous-cell carcinoma (SCC). Patient data were collected from 2016 to 2020 and the cervical specimens were separated by histopathology into four disease stages: endocervicitis (normal group), LSIL, HSIL, and SCC. In the four stages of cervical cancer progression, there were 105 cases (55.26%) in endocervicitis, 36 cases (40.45%) in LSIL, 67 cases (56.78%) in HSIL and 21 cases (56.76%) in SCC of single high-risk HPV infection (Table 1). In addition, 52 cases (27.37%) had endocervicitis, 39 cases (43.82%) in LSIL, 31 cases (26.27%) had HSIL and 10 cases (27.03%) had SCC of 2 or more high-risk HPV subtypes (Table 1). In our data, HPV16, 52, 56 in high-risk HPV type, HPV53 in middle-risk HPV type and HPV81 in low-risk HPV type were found to be the main HPV infection types (Fig. 1A). We also found that age distribution was associated with SIL development and SCC initial formation (Fig. 1B). The average age of patients with SCC was 52.59 years (41–68 years). Compared with SCC patients, SIL patients were much younger, with an average age of 45.78 years (22–70 years), of which the average age of patients with LSIL was 44.33 years (22–69 years) and the average age of patients with HSIL was 46.88 years (27–70 years).

**Table 1:** Infection of different types of HPV in patients with endocervicitis, SIL and CSCC.

HPV types		Endocervicitis (*n* = 190)		LSIL (*n* = 89)		HSIL (*n* = 118)		CSCC (*n* = 37)	
		*n*	%	*n*	%	*n*	%	*n*	%
HR-HPV	Single type	105	55.26	36	40.45	67	56.78	21	56.76
	Multiple infections	52	27.37	39	43.82	31	26.27	10	27.03
MR-HPV	Single type	16	8.42	10	11.24	9	7.63	6	16.22
	Multiple infections	22	11.58	17	19.10	10	8.47	3	8.11
LR-HPV	Single type	12	6.32	2	2.25	11	9.32	0	0.00
	Multiple infections	10	5.26	7	7.87	11	9.32	2	5.41

*HR-HPV* high risk human papillomavirus (including 16, 18, 31, 33, 35, 39, 45, 51, 52, 56, 58, 59, 68, 73, and 82 type), *MR-HPV* middle risk human papillomavirus, *LR-HPV* low-risk human papillomavirus.

The results above indicated that high-risk HPVs were the main driving factors that facilitate SIL development and promote SCC initial formation, and persistence of HPV infection was another important factor for cervical cancer emergence.

### The alteration of ferroptotic genes was associated with SIL-to-SCC transitions

**Fig. 2:**
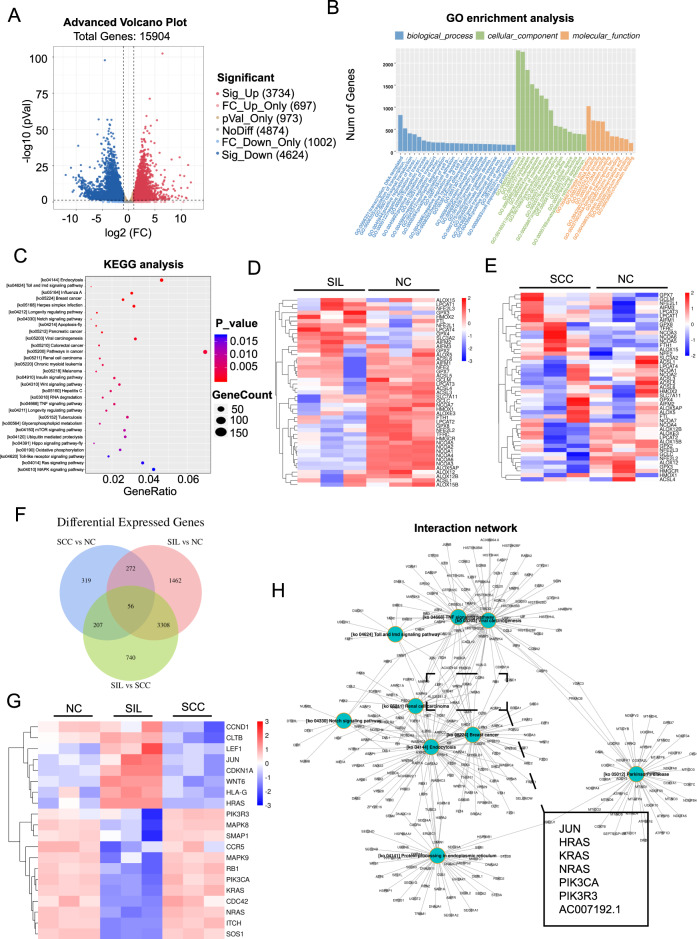
The ferroptotic genes changed through SIL-to-SCC transitions. **A** RNA-seq data of three pairs of SIL tissues and matched adjacent tissues was used to identify DEGs by Volcano plot. Red and blue dots, respectively indicated the upregulated and downregulated genes. **B** DEGs enriched in biological process, cellular component or molecular function were shown in GO enrichment analysis. The height of the bar represents the enrich gene number. **C** DEGs enriched in pathway were shown in KEGG analysis. The abscissa represents the gene ratio, which is calculated as “input gene number”/“background gene number”. **D** Genes enriched in ferroptotic pathway were shown in the heat map. Data was normalized by logarithm of 2. **E** Genes enriched in ferroptotic pathway from our previous RNA-seq data [18] were shown in the heat map. Data was normalized by logarithm of 2. **F** DEGs enriched that both changed in normal to SIL transition and SIL to SCC transition were shown in the Venn diagram. Data was normalized by logarithm of 2. **G** DEGs enriched were shown in the heat map. **H** Global view of DEGs enriched in KEGG pathway were shown in interaction network.

We first performed RNA-seq to identify genes and pathways affected in normal-to-SIL transitions with three pairs of SIL tissues and matched adjacent tissues. A volcano plot showed that significant transcriptional changes were detected with 8358 (52.55%) differentially expressed genes (DEGs) out of 15,904 detectable genes (|log2FC | >1 and *p* < 0.05), of which 3734 were upregulated and 4624 were downregulated in the SIL tissue (Fig. 2A). GO enrichment analysis revealed that the enriched clusters were related to signal transduction and regulation of transcription in biological processes and metal ion binding and RNA binding in molecular functions, as shown in Fig. 2B. KEGG analysis revealed that the enriched clusters were related to endocytosis, viral carcinogenesis and oxidative phosphorylation, as shown in Fig. 2C. To validate whether the DEGs were associated with ferroptosis, we measured the expression of ferroptosis-related genes in each cluster and performed hierarchical Euclidean distance analysis. The heatmap results showed that anti-ferroptotic genes decreased in SIL tissues, indicating that ferroptosis occurred in SILs (Fig. 2D). This result reminded us to explore whether anti-ferroptotic genes decreased in SCC tissues similar to SILs. With reanalysis of our previous RNA-seq data [18], no significant difference was found between cervical cancer tissues and adjacent tissues (Fig. 2E). Moreover, we examined the expression levels of ferroptosis-related genes in cervical cancer tissues and adjacent samples from TCGA RNA-seq data from 317 documented patients. We found that anti-ferroptotic genes such as GCLM, GCLC, GSR, and GPX4 were increased (Table S2). These results suggested that the alteration of ferroptotic genes was associated with SIL-to-SCC transitions. Next, we combined RNA-Seq data of SILs and previous data of SCC samples to explore which signaling pathway promoted the development from SIL to SCC. As shown in Fig. 2F, a Venn diagram and heatmap revealed 56 DEGs that both changed in the normal to SIL transition and the SIL to SCC transition. KRAS, NRAS and HRAS, referred to as oncogenic RAS, gained our attention from the viral carcinogenesis pathway, breast cancer pathway and renal cell carcinoma pathway (Fig. 2G, H). We found that KRAS was decreased in LSILs and HSILs, but increased in the SCC stage (Fig. 2G, H).

These results suggested that ferroptosis may regulate tumorigenesis through the RAS signaling pathway during the SIL to SCC transition.

### Low-dose erastin-induced ferroptosis does not affect cervical cancer cell viability

**Fig. 3:**
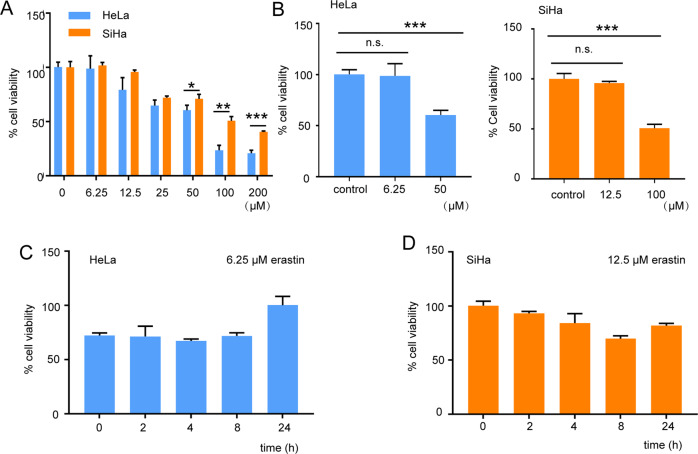
Mild ferroptosis does not affect cervical cancer cell viability. **A**, **B** The HeLa cells and SiHa cells were cultured for the indicated periods. The cell viability of HeLa cells and SiHa cells treated with indicated concentration of erastin for 24 h was detected with a CCK8 biochemical detection assay (*n* = 3). **C** The HeLa cells were treated with 6.25 μM erastin for indicated time. The cell viability was detected with a CCK8 biochemical detection assay (*n* = 3). **D** The SiHa cells were treated with 12.5 μM erastin for indicated time. The cell viability was detected with a CCK8 biochemical detection assay (*n* = 3). All data are from three independent experiments. The data are presented as the mean ± SD values (*n* ≥ 3). *P < 0.05; **P < 0.01, ***P < 0.001.

To explore the effect of ferroptosis on SIL development, HeLa cells (cervix epithelial cells from adenocarcinoma patients) and SiHa cells (cervix epithelial cells from squamous cell carcinoma patients) were treated with erastin (a classical ferroptosis inducer). We found that SiHa cells were less sensitive to erastin than HeLa cells (Fig. 3A). The viability of SiHa cells did not significantly decline until the concentration of erastin reached 100 μM (high dose). We also verified their cell viability with a low dose (12.5 μM) of erastin treatment and found that it was not significantly changed (Fig. 3B). A similar result was found in HeLa cells: low-dose (6.25 μM) erastin treatment did not affect HeLa cell viability (Fig. 3B). As shown in Fig. 3C, D, when the cells were treated with a low dose of erastin, the viability of SiHa and HeLa cells decreased briefly and quickly recovered. This result reminded us to verify whether a low dose of erastin-induced ferroptosis affects tumorigenesis.

### Low-dose erastin-induced ferroptosis increases KRAS expression in cervical cancer cells

**Fig. 4:**
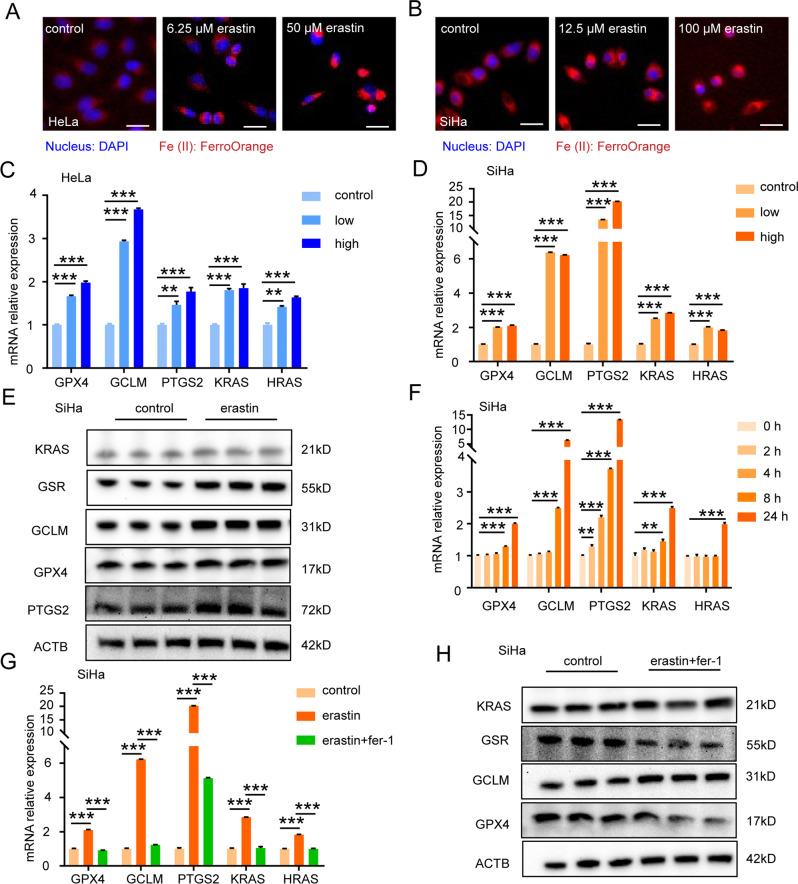
Mild ferroptosis increases KRAS expression. **A**, **B** The HeLa cells and SiHa cells were treated with indicated concentration of erastin for 24 h. The HeLa cells and SiHa cells were fixed and stained with FerroOrange (red) and DAPI (blue) to identify Fe-(II). Scale bar, 50 μm. **C**, **D** qPCR was used to analyze the mRNA levels of GPX4, GCLM, PTGS2, KRAS, and HRAS in the HeLa cells and SiHa cells treated with high or low dose of erastin for 24 h (*n* = 3). **E** Lysates of SiHa cells treated with or without low dose of erastin for 24 h were immunoblotted for KRAS, GSR, GCLM, GPX4, PTGS2, and ACTB (*n* = 3). The data are normalized to the ACTB control. **F** qPCR was used to analyze the mRNA levels of GPX4, GCLM, PTGS2, KRAS, and HRAS in the SiHa cells treated with low dose of erastin for indicated time (*n* = 3). **G** qPCR was used to analyze the mRNA levels of GPX4, GCLM, PTGS2, KRAS, and HRAS in the SiHa cells treated with or without low dose of erastin and fer-1 for 24 h (*n* = 3). **H** Lysates of SiHa cells treated with or without low dose of erastin and fer-1 for 24 h were immunoblotted for KRAS, GSR, GCLM, GPX4, PTGS2, and ACTB (*n* = 3). The data are normalized to the ACTB control.All data are from three independent experiments. The data are presented as the mean ± SD values (*n* ≥ 3). **P < 0.01, ***P < 0.001.

RAS is a well-known oncoprotein that regulates growth and differentiation in cancer cells. KRS, HRAS, and NRAS are three subunits of RAS that have been reported to be associated with tumorigenesis. To determine whether ferroptosis can affect tumorigenesis, HeLa cells, and SiHa cells were treated with low or high doses of erastin, and RAS-related genes were detected. Using an Fe-(II) detecting probe in HeLa cells and SiHa cells, the fluorescence images showed an increased fluorescence intensity in the cells treated with a low or high dose of erastin compared to that in untreated cells (Fig. 4A, B). Similarly, compared to the control groups, the mRNA expression of PTGS2 (a ferroptosis marker, but not the driving factor of ferroptosis [13]) increased in both the low- and high-dose treatment groups, indicating that ferroptosis occurred (Fig. 4C, D). We found that *GCLM* and *GPX4* (two antiferroptosis genes) increased in the low- and high-dose erastin treatment groups, indicating that HeLa cells and SiHa cells presented an antiferroptotic effect in response to erastin-induced ferroptosis (Fig. 4C, D). Similar protein expression results were found in SiHa cells, in which KRAS, GPX4, GSR, and PTGS2 were increased in the erastin treatment group compared to the control group (Fig. 4E). These results indicated that low-dose erastin treatment caused both anti-ferroptotic effects and tumorigenesis. To explore whether ferroptosis or anti-ferroptosis can increase KRAS, we next treated SiHa cells with a low dose of erastin at planned time points to observe dynamic changes in target genes. We found that both *KRAS* and anti-ferroptotic genes (*GCLM* and *GPX4*) increased after 8 h of treatment, while *PTGS2* increased after 2 h of treatment in SiHa cells, indicating that the anti-ferroptotic effect emerged after ferroptosis occurred (Fig. 4F). We also found that *GCLM* significantly increased (2.50-fold), while *KRAS* increased slightly (1.45-fold), indicating that the anti-ferroptotic effect occurred earlier than *KRAS* upregulation (Fig. 4F). Furthermore, when we blocked ferroptosis in SiHa cells with fer-1 (a ferroptosis inhibitor), both the mRNA and protein expression levels of KRAS and GPX4 decreased (Fig. 4G, H). These results indicated that ferroptosis resistance was stimulated by mild ferroptosis and promoted tumorigenesis by enhancing RAS signaling.

Taken together, our results showed that low-dose erastin treatment in cervical cancer cells not only induced ferroptosis but also led to ferroptosis resistance by increasing GCLM and GPX4, finally enhancing KRAS expression, indicating that mild ferroptosis induced anti-ferroptotic effects and thereby promoted tumorigenesis in cervical cancer cells.

### A cascade of ferroptosis-related gene expression changes at the stage of cervical SIL progression

**Fig. 5:**
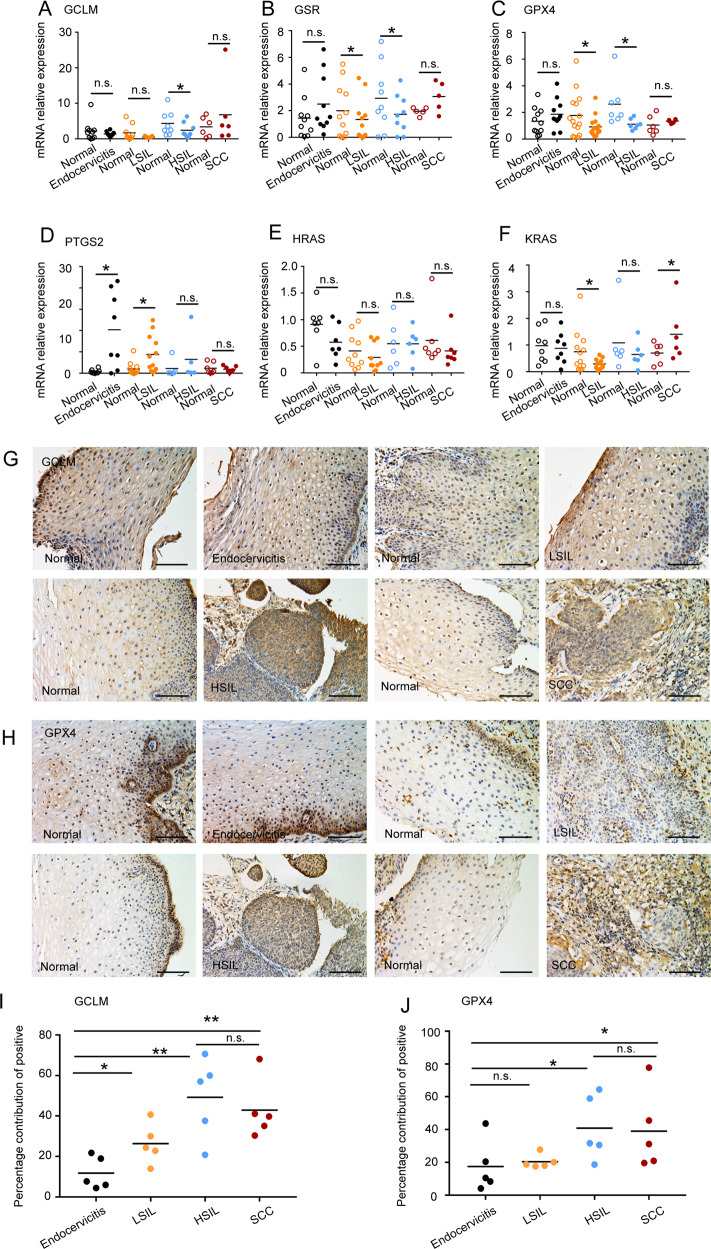
Ferroptosis-related gene expression changes at the stage of cervical SIL Progression. **A**–**F** qPCR was used to analyze the mRNA levels of GPX4, GCLM, GSR, PTGS2, KRAS, and HRAS in endocervicitis, LSIL, HSIL, SCC, and matched adjacent normal tissues. **G**, **H** Tissue sections in endocervicitis, LSIL, HSIL, SCC were immunostained with anti-GCLM or anti-GPX4 using immunohistochemistry. Scale bar, 200 μm. **I** Percentage contribution of positive of GCLM in endocervicitis, LSIL, HSIL, SCC related to **G**. **J** Percentage contribution of positive of GPX4 in endocervicitis, LSIL, HSIL, SCC related to **H**. All data are from three independent experiments. The data are presented as the mean ± SD values (*n* ≥ 3). *P < 0.05; **P < 0.01, ***P < 0.001, n.s.: not significant.

The in vitro results above showed that the antiferroptotic effect promoted KRAS-mediated tumorigenesis in cervical cancer cells, and our previous RNA-seq data also indicated that a series of ferroptosis-related genes changed in the SIL to SCC transition. To verify this hypothesis in vivo, 47 cervical specimens including endocervicitis, LSIL, HSIL, and SCC, together with their adjacent normal tissues were used to detect the expression of ferroptosis-related genes by qPCR. Anti-ferroptotic genes such as *GCLM*, *GSR* and *GPX4* were decreased in LSIL and HSIL compared to their matched adjacent tissues, while no significant difference was found between cervical cancer tissues and adjacent samples (Fig. 5A–C). We also found that *PGTS2* expression was higher in endocervicitis and LSILs than in their matched adjacent tissues, further supporting our previous results that ferroptosis occurs in the stage of LSIL and anti-ferroptosis occurs in the stage of SCC (Fig. 5D). We also detected the protein expression levels of GCLM and GPX4 by immunohistochemistry (IHC). The IHC results showed that the positive sites of GCLM and GPX4 were not only found in SCC samples but also observed at the lesion site in some HSILs, although they presented at low levels in peripheral to lesions. This indicated that anti-ferroptotic effects also occurred in the HSIL stage in addition to the SCC stage (Fig. 5G, H). We further found that KRAS, not HRAS, was decreased in LSILs compared to their matched adjacent tissues (Fig. 5E, F). However, when most lesion sites presented anti-ferroptotic effects, as in SCCs, KRAS increased significantly (Fig. 5F).

Taken together, HR-HPV infected SIL presented ferroptosis and reduced KRAS expression, while SCC exhibited anti-ferroptotic effects and increased KRAS expression.

### Intercellular iron presents high levels throughout HPV-induced cervical cancer progression

**Fig. 6:**
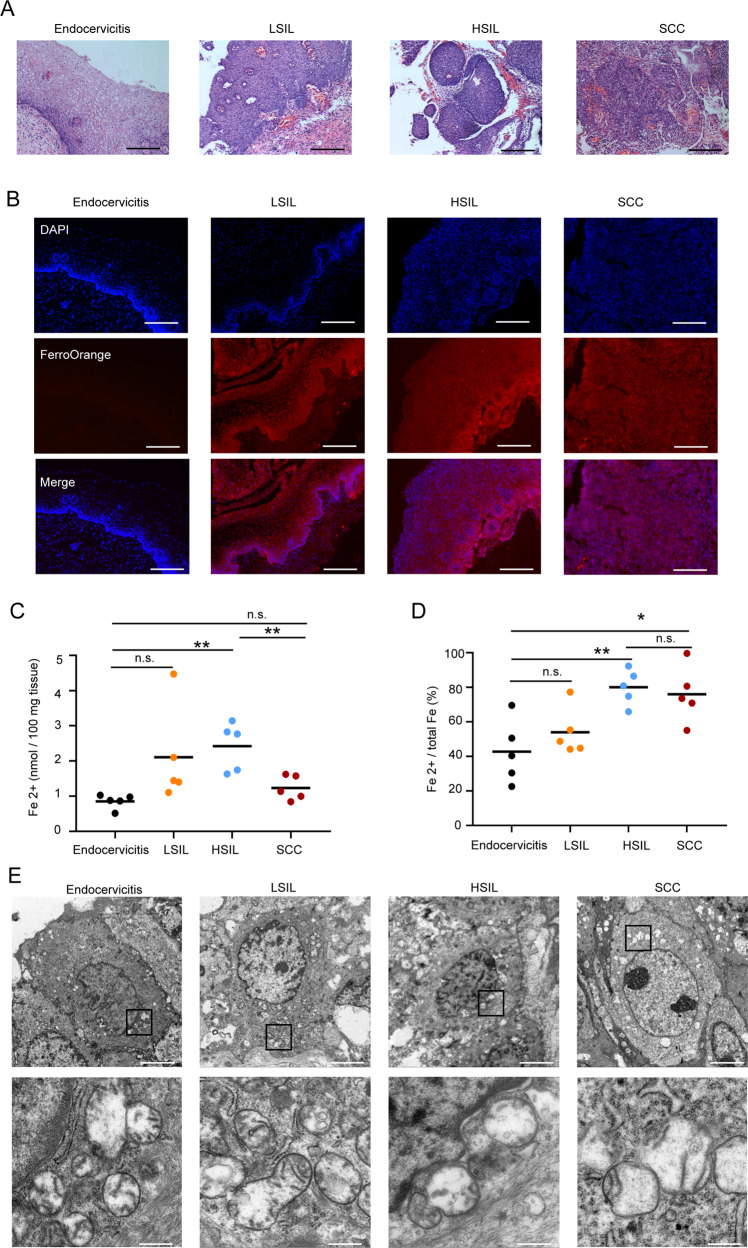
High level of intercellular iron and abnormal mitochondria morphology present in cervical SIL progression. **A** The histological sections were stained with HE dyes to indicate the morphology of lesion sites in endocervicitis, LSIL, HSIL, and SCC. Scale bar, 200 μm. **B** The freezing tissue slices were fixed and stained with FerroOrange (red) and DAPI (blue) to identify Fe-(II) in endocervicitis, LSIL, HSIL, and SCC. Scale bar, 200 μm. **C**, **D** The concentration of Fe-(II) and Fe-(II)/total Fe ratio in endocervicitis, LSIL, HSIL, and SCC was detected with an Iron Assay Kit (*n* = 5). **E** TEM images of mitochondrial morphology in endocervicitis, LSIL, HSIL, and SCC. Scale bar, 5 μm (insets, 500 nm) All data are from three independent experiments. The data are presented as the mean ± SD values (*n* ≥ 3). *P < 0.05; **P < 0.01, n.s.: not significant.

Next, we stained cervical tissues with FerroOrange to detect intercellular Fe-(II) levels for further verification of the ferroptosis phenotype. The cervical specimens were separated by histopathology into four disease stages: normal, LSIL, HSIL, and SCC (Fig. 6A). As shown in Fig. 6B, fluorescence indicating Fe-(II) accumulation was found in cervical basal cells of LSIL tissues and in squamous cells or basal cells of HSIL tissues, while fluorescence was scarcely found in normal specimens. Fluorescence was also found in the whole SCC samples, but its intensity was lower than that in HSIL tissues. In addition, Fe-(II) was measured with an iron biochemical detection kit and a similar result was found. As shown in Fig. 6C, Fe (II) levels were increased in the stage of LSIL to SCC compared with those in normal specimens. The Fe-(II) level was higher in HSIL than SCC, while the Fe-(II)/total Fe ratio was similar between the two groups (Fig. 6C, D).

These results showed that HP-HPV-induced intercellular Fe-(II) content enrichment in SIL and SCC, indicating an association between SCC pathogenesis and ferroptosis.

### Reduced mitochondrial cristae and impaired outer mitochondrial membranes are present in cervical SIL progression

As a previous study described, ferroptosis is distinct from apoptosis, necrosis, and autophagy in morphological characteristics and biochemical features [19]. The most important characteristics of ferroptosis induced by erastin are reduced or absent mitochondrial cristae and increased density of outer mitochondrial membranes in morphological characteristics [19]. Similarly, we found that the number of mitochondrial cristae was reduced in LSILs and even rarely observed in HSILs, but the number of mitochondrial cristae in SCC tissues was increased compared with that in HSIL (Fig. 6E). In addition, vacuolization indicating mitochondrial membrane damage was observed in LSILs and HSILs. Likewise, we found that the outer mitochondrial membrane boundaries are blurred in LSIL to SCC stages (Fig. 6E). These results suggested a phenotype of mitochondria similar to ferroptosis in LSILs and HSILs.

Taken together, the results above showed that ferroptosis occurred in both LSILs and HSILs and that antiferroptosis formed in SCCs. Our in vitro and in vivo results indicated that potential primary tumor lesion sites formed anti-ferroptotic effects and thereby promoted tumorigenesis in response to ferroptosis induced by persistent HR-HPV infection.

## Discussion

Most patients with SCC are reported to be HPV positive. Persistent HPV infection is identified as the main reason for cervical cancer emergence, of which HR-HPVs act as its main aspect [20]. A previous meta-analysis of HPV type distribution related to SIL development in China reported that HPV52 is the most common HPV type in LSIL, followed by HPV16 and HPV58; HPV16 is the most common HPV type in HSIL, followed by HPV58 and HPV52 [21]. HPV16, HPV58, and HPV33 are reported to have higher carcinogenic risk among other HPV types in southwestern China [22]. Similarly, in our study, we analyzed HPV genotypes of the patients diagnosed with HPV from 2016 to 2020 in our hospital related to different stages of cervical lesions. We found that HPV52, HPV16, and HPV58 were the top three common HR-HPV types, although HPV 56 was the main genotype of HP-HPV infection in SCC patients. Our results indicated that the SIL to SCC transition was conducted by certain HPV types.

HPV infection leads to a series of changes in cell proliferation, cell death evasion, and genomic instability by influencing lipid metabolism and oxidative stress and thereby determines cell fate [12]. It was reported that HPV-positive cervical cancer cells presented metabolic reprogramming by promoting SIRT expression [23]. Increasing FA metabolism and oxidative phosphorylation by expressing SIRT in SiHa cells and C33a cells promoted the invasion and metastasis of cervical cancer cells [23]. Head and neck squamous cell cancer (HNSCC) overexpressing E6 protein from HPV16 and HPV18 produced more ROS than HPV-negative HNSCC by inducing mitochondrial metabolism [24]. Oxidative phosphorylation is reported to be a preferred method to obtain energy in HPV-positive HNSCC [25]. HPV-related cancer cells are capable of lipid peroxidation clearance for the maintenance of cellular life [14]. Similarly, our results showed that *GPX4*, an anti-lipid peroxidation gene, increased in SCC and erastin-induced cervical cancer cell lines suggesting that cervical cancer cells were able to clear excess lipid peroxide, and thereby to maintain cancer cell survival.

Moreover, iron-dependent lipid peroxidation is well known as the main feature of ferroptosis. It was reported that inducing iron deprivation by desferrioxamine (DFO) could inhibit the growth of HPV-positive carcinoma cells [26]. E6/E7 oncogene expression was repressed by treatment with the iron chelator ciclopirox (CPX) in HPV-positive carcinoma cells [27]. Similarly, we found that tissues presented high levels of total Fe through the SIL to SCC transition. We also found a decrease in Fe-(II) levels in SCC compared to SIL, indicating that cervical cancer cells may undergo metabolic reprogramming to cope with the ferroptosis environment. In addition, it was reported that HNSCC cells expressing E6 and E7 oncoproteins from HPV16 were sensitive to erastin-induced ferroptosis [28]. We showed a similar result: cervical tissue infected by HR-HPV was sensitive to ferroptosis in the SIL stage, and we further found that SCC tissue and cell lines were resistant to ferroptosis in response to persistent ferroptosis.

Ras is an oncogene that modulates many downstream pathways controlling cell proliferation, survival and other aspects of cell behavior [29]. KRAS, HRAS and NRAS, the three subsets of RAS, are well known to be activated by mutation in nearly a quarter of all human cancers, of which KRAS plays a key role in driving tumorigenesis [30]. KRAS functions as a molecular switch to regulate GDP-GTP cycling and is involved in the progression of normal pancreatic tissue to PDAC, and KRAS mutation is considered a driver for PDAC initial emergence [31–33]. Increased HRAS expression levels promote gastric carcinoma cell aggressiveness [34]. Resent research has reported that the blockade of Nrf2/HO-1 signaling promotes RSL3-induced ferroptosis in KRAS mutant colorectal cancer [35]. However, it was reported that KRAS mutations were rarely found in CSCC but were frequently observed in cervical adenocarcinomas, indicating that KRAS mutations in CSCC were uncommon [36]. Our in vivo results demonstrated that *KRAS* was decreased in SIL but increased in SCC. In our in vitro experiments, following a classic method [37], we treated HeLa and SiHa cells with erastin to induce cell death. To simulate the ferroptosis effect in vivo, we also used a low dose of erastin that induced a mild ferroptosis effect without causing most cell death. Our results showed that the high level of KRAS expression in carcinoma cells was due to an antiferroptotic effect, from which carcinoma cells respond to a low dose of erastin-induced ferroptosis.

Our study investigated the relationship between ferroptosis and HPV-induced cervical diseases, and explored its underlying mechanism: (i) Ferroptosis occurs in LSIL with HR-HPV infection; (ii) persistent ferroptosis promotes SIL development and causes anti-ferroptotic effects; and (iii) lesions with anti-ferroptotic effects increase KRAS to promote tumorigenesis in SCC.

### Limitations of the study

Although ferroptosis is proposed to be a novel therapeutic target in some types of cancer, the underlying molecular mechanism of ferroptosis in SIL to SCC transition is not fully understood. We acknowledge that the present study has several limitations, including the factors that regulate the formation of initial cancer cells in SIL. In addition, we just investigated HR-HPV infected SIL development and SCC formation, but which certain types of HPV, including HPV52, HPV16 and HPV58, affected SCC formation are remained unexplained. Future studies will focus on the regulation factors of initial cancer cells formation and to clarify the underlying function of certain types of HPV in SCC formation.

## Materials and methods

### Chemicals and antibodies

Erastin and Ferrostatin-1 were purchased from Selleckchem (Shanghai, China). Iron Assay Kit (I291) and FerroOrange (#F374) were purchased from Dojindo Laboratories (Kumamoto, Japan). Antibodies against GPX4 (#A1933), GSR (#A4566), ACTB (#AC026), GCLM (#A13989), PTGS2 (#A1253), KRAS (#A12704) as well as secondary HRP-conjugated goat anti-rabbit IgG (#AS014) were from ABclonal (Wuhan, China).

### Sample collection and preparation

Cervical specimens including endocervicitis, LSIL, HSIL, and SCC, together with their adjacent normal tissues were collected from patients who underwent surgical operation from January 2021 to August 2021 in Department of Obstetrics and Gynecology, The Affiliated Jiangning Hospital with Nanjing Medical University. Specimens were frozen in liquid nitrogen immediately after operation and stored at −80 °C until extraction. All samples were confirmed by histopathological examination.

### Cell culture

The SiHa and Hela cells were cultured in Dulbecco’s modified Eagle’s medium (DMEM, Biochannel, China) supplemented with 10% fetal bovine serum (FBS, Biochannel, China) in a humidified atmosphere containing 5% CO_2_ at 37 °C. Before the treatment of ferroptosis inducer or inhibitor, the Hela cells and SiHa cells were grown on coverslips in 24-well plates overnight.

### Histological detection of labile Fe-(II)

The method of histological detection of labile Fe-(II) was followed as described [38, 39]. The 8 μm thickness frozen sections were immobilized with phosphate-buffered formalin for 1 min. Then frozen sections were stained in FerroOrange (RhoNox-4) (used at 1:1000 dilution) in 10 mM PBS (pH 7.4) for 30 min at 37 °C in a dark chamber and imaged immediately. Fluorescent microscope was used with ZEN software to acquire high-quality images.

### Tissue iron detection

Tissues were washed with 1 mL ice-cold PBS twice, then were fully grinded with assay buffer according to an Iron Assay Kit protocol (DOJINDO, Japan). The samples were divided into three parts: Fe-(II) detection, total Fe detection and blank control. The sample for total Fe detection was added with reducer solution and the rest samples were added with equal volumes assay buffer. All samples were incubated for 10 min at 37 °C, then transferred into 96-well plate and continued to incubated for 60 min at 37 °C. Automatic biochemical analyzer was used to detect absorbance at 593 nm.

### Western blot analysis

The samples obtained from the cells or tissues lysed with RIPA buffer (Beyotime, China) were separated by 10% SDS–PAGE (GenScript, China) and transferred onto PVDF membranes (Merck, Germany). The membranes were blocked with Western Blocking Solution (Beyotime, China) for 1 h and then incubated with primary antibody overnight at 4 °C. The next day, the membranes were incubated with secondary antibodies for 2 h. The bands were visualized by a chemiluminescence method.

### Total RNA isolation and real-time fluorescence quantitative PCR analysis

Total RNA was extracted by TRIzol reagent (Invitrogen, USA), and cDNA was synthesized using a HiScript II one step RT–PCR kit (Vazyme, China). The quantitative real-time PCR (qPCR) was performed on a StepOne Plus system (Applied Biosystems, USA) with ChamQ SYBR qPCR Master Mix (Vazyme, China). The primers used to amplify the specific genes are listed in the Table S1.

### Immunohistochemistry

The paraffin sections were subjected to gradient ethanol dewaxing and followed by antigen repair. Then, the samples were blocked with 10% goat serum (#005-000-121, Jackson Immuno-Research, USA) for 1 h at room temperature, and incubated overnight with primary antibodies at 4 °C: antibodies against GPX4 and GCLM were used at 1:100 dilution. The next day, the samples were incubated with secondary antibodies for 1 h at 37 °C: HRP-conjugated goat anti-rabbit IgG (used at 1:200 dilution, ABclonal). Images were acquired by microscopy.

### Ethical statements

The clinical study was approved by the Ethics Committee of Jiangning Hospital (No. 2021-03-020-K01). All patients signed an informed consent form prior to the study.

### Statistical analysis

All data are presented as the mean ± SD. Student’s *t* test was used for comparisons between two independent sample groups, one-way analysis of variance (ANOVA) was used for single-factor comparisons among multiple groups, and two-way ANOVA was used for two-factor comparisons among multiple groups; *P* < 0.05 was regarded as significant.

## Supplementary information

TABLE S1

TABLE S2

western blots data

## Data Availability

All data generated or analyzed during this study are included in this published article.
