# Supplementary material for: Persistent ferroptosis promotes cervical squamous intraepithelial lesion development and oncogenesis by regulating KRAS expression in patients with high risk-HPV infection
Source: Cell Death Discov. 2022 Apr 14;8:201. doi: 10.1038/s41420-022-01013-5 (PMC9010439; doi:10.1038/s41420-022-01013-5)
Supplement: Supplementary file 1 — TABLE S1 [file 41420_2022_1013_MOESM1_ESM.docx]

**Table S1** Primer sequences.

| Gene |  | primer |
| --- | --- | --- |
| ACTB (human) | Forward | 5′- GCCGAGGACTTTGATTGC-3′ |
|  | Reverse | 5′- CCTGTGTGGACTTGGGAGA-3′ |
| GCLM (human) | Forward | 5′-ACAAGCTAAAAGAAGGGGTTC-3′ |
|  | Reverse | 5′-GACGAAAGAATATCTGCCTCA-3′ |
| GPX4 (human) | Forward | 5′- CAGTTCGGGAAGCAGGAG-3′ |
|  | Reverse | 5′- GCCCTTGGGTTGGATCTT-3′ |
| GSR (human) | Forward | 5′-CACTTGCGTGAATGTTGG-3′ |
|  | Reverse | 5′-TAGGCATCCCGCTTTTC-3′ |
| HRas (human) | Forward | 5′-TGCCATCAACAACACCAAG-3′ |
|  | Reverse | 5′-CCTGCCGAGATTCCACA-3′ |
| KRas (human) | Forward | 5′-TGGCGTAGGCAAGAGTG-3′ |
|  | Reverse | 5′-TTGACCTGCTGTGTCGAG-3′ |
| PTGS2 (human) | Forward | 5′-GTATGAGTGTGGGATTTGACC-3′ |
|  | Reverse | 5′-TGTGTTTGGAGTGGGTTTC-3′ |
