# Supplementary material for: Persistent ferroptosis promotes cervical squamous intraepithelial lesion development and oncogenesis by regulating KRAS expression in patients with high risk-HPV infection
Source: Cell Death Discov. 2022 Apr 14;8:201. doi: 10.1038/s41420-022-01013-5 (PMC9010439; doi:10.1038/s41420-022-01013-5)
Supplement: Supplementary file 2 — TABLE S2 [file 41420_2022_1013_MOESM2_ESM.docx]

**Table S2**

Ferroptosis gene expression in Cervical Cancer from TCGA, stratified according to their sample types.

|  | Solid Tissue Normal | Primary Tumor | Fold | p value |
| --- | --- | --- | --- | --- |
|  | (n=3) | (n=317) | Solid Tissue Normal v.s. Primary Tumor | (FDR) |
| **Aminoacid transporters** | | | | |
| SLC7A11 | 0.0448 | 0.780 | Up | 0.2490 |
| SLC3A2 | 5.15 | 5.43 | Up | 0.5153 |
|  | | | | |
| **Antioxidant defenses/Lipid metabolism** | | | | |
| GPX4 | 6.44 | 6.48 | Up | 0.4760 |
| AIFM2 | 1.97 | 2.33 | Up | 0.8012 |
| ACSL4 | 3.38 | 2.82 | Down | 0.1046 |
| ALOX5 | 1.51 | 2.32 | Up | 0.2893 |
| ALOX12 | 0.625 | 0.858 | Up | 0.4351 |
| ALOX15 | 0.0467 | 0.295 | Up | 0.4352 |
| ALOX15B | 0.0624 | 1.12 | Up | 0.1687 |
| NFE2L2 | 4.88 | 4.99 | Up | 0.9518 |
| HMOX1 | 2.87 | 3.66 | Up | 0.1990 |
| NFS1 | 2.05 | 2.46 | Up | 0.02823 |
| GSS | 4.28 | 5.64 | Up | 4.294e-06 |
| GSR | 3.87 | 4.75 | Up | 0.3517 |
| GCLM | 1.73 | 2.71 | Up | 0.07654 |
| GCLC | 2.20 | 2.64 | Up | 0.5909 |
|  | | | | |
| **Iron/autophagy** | | | | |
| TFRC | 3.47 | 5.16 | Up | 4.473e-03 |
| FTH1 | 7.53 | 7.58 | Up | 0.4118 |
| NCOA4 | 5.10 | 5.27 | Up | 0.8115 |
| SQSTM1 | 5.24 | 4.48 | Down | 0.5065 |

RNA expression is given as median log2 (fpkm+1). *p* values obtained with Wilcoxon-Mann-

Whitney test after FDR correction. Note that the genes with significantly different expression levels are in bold.
