# Supplementary material for: Persistent ferroptosis promotes cervical squamous intraepithelial lesion development and oncogenesis by regulating KRAS expression in patients with high risk-HPV infection
Source: Cell Death Discov. 2022 Apr 14;8:201. doi: 10.1038/s41420-022-01013-5 (PMC9010439; doi:10.1038/s41420-022-01013-5)

GCLM related to Fig. 4e


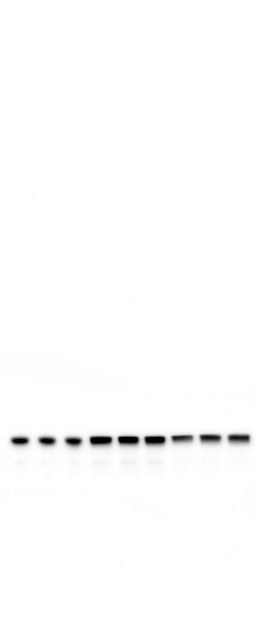


GSR related to Fig. 4e


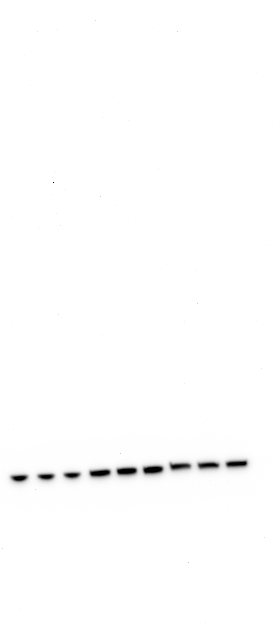


PTGS2 related to Fig. 4e


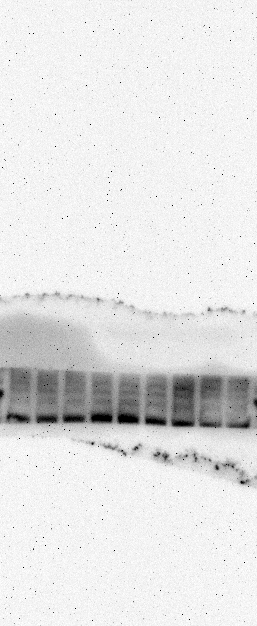


KRAS and GPX4 related to Fig. 4e


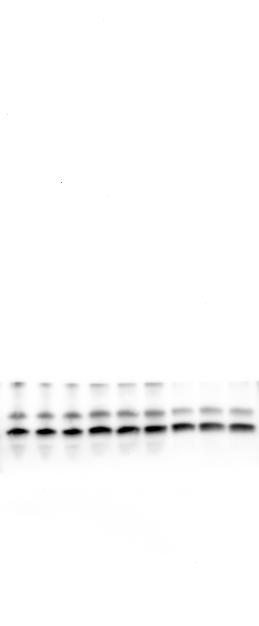


ACTB related to Fig. 4e


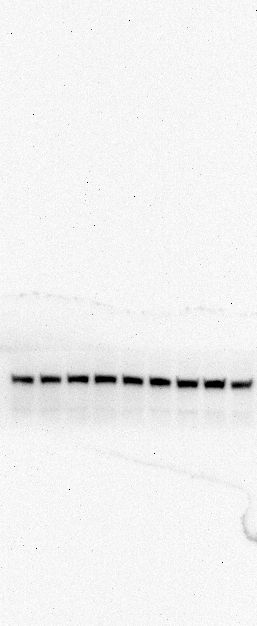


GCLM related to Fig. 4h


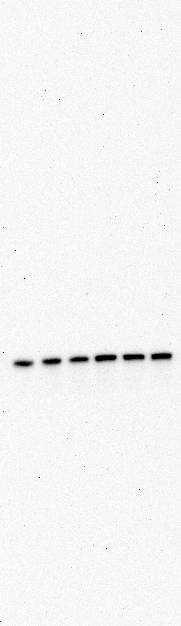


GSR related to Fig. 4h


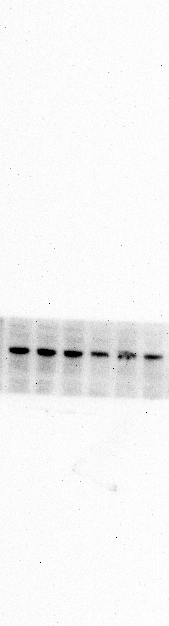


KRAS related to Fig. 4h


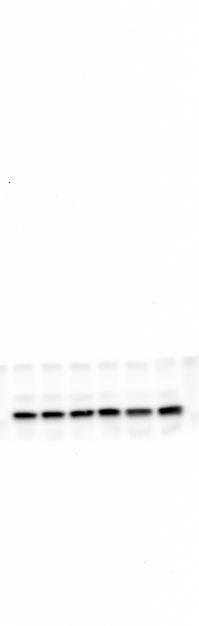


GPX4 related to Fig. 4h


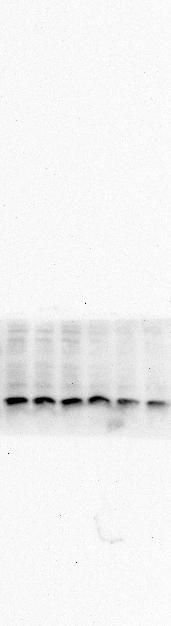


ACTB related to Fig. 4h


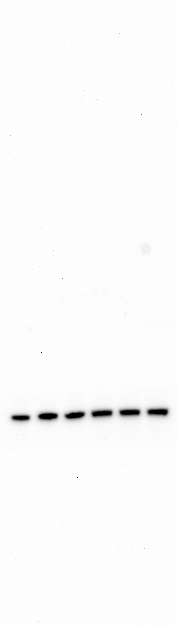

Supplement: Supplementary file 3 — western blots data [file 41420_2022_1013_MOESM3_ESM.docx]
